# Gain of Function for the *SCN1A*/hNa_v_1.1-L1670W Mutation Responsible for Familial Hemiplegic Migraine

**DOI:** 10.3389/fnmol.2018.00232

**Published:** 2018-07-09

**Authors:** Sandra Dhifallah, Eric Lancaster, Shana Merrill, Nathalie Leroudier, Massimo Mantegazza, Sandrine Cestèle

**Affiliations:** ^1^Université Côte d’Azur, CNRS UMR 7275, INSERM, IPMC, Valbonne, France; ^2^Department of Neurology, University of Pennsylvania, Philadelphia, PA, United States

**Keywords:** migraine with aura, sodium channels, GABAergic neurons, cortical spreading depression, epilepsy

## Abstract

The *SCN1A* gene encodes for the voltage-dependent Na_v_1.1 Na^+^ channel, an isoform mainly expressed in GABAergic neurons that is the target of hundreds of epileptogenic mutations. More recently, it has been shown that the *SCN1A* gene is also the target of mutations responsible for familial hemiplegic migraine (FHM-3), a rare autosomal dominant subtype of migraine with aura. Studies of these mutations indicate that they induce gain of function of the channel. Surprisingly, the mutation L1649Q responsible for pure FHM-3 showed a complete loss of function, but, when partially rescued it induced an overall gain of function because of modification of the gating properties of the mutant channel. Here, we report the characterization of the L1670W *SCN1A* mutation that has been previously identified in a Chinese family with pure FHM-3, and that we have identified also in a Caucasian American family with pure FHM-3. Notably, one patient in our family had severe neurological deterioration after brain radiation for cancer treatment. Functional analysis of L1670W reveals that the mutation is responsible for folding/trafficking defects and, when they are rescued by incubation at lower temperature or by expression in neurons, modifications of the gating properties lead to an overall gain of function. Therefore, L1670W is the second mutation responsible for FHM-3 with this pathophysiological mechanism, showing that it may be a recurrent mechanism for Na_v_1.1 hemiplegic migraine mutations.

## Introduction

Migraine is a chronic neurovascular disorder characterized by recurrent disabling attacks of severe, unilateral, throbbing headache with autonomic dysfunctions lasting up to 3 days (Diener et al., 2012; Goadsby et al., 2017). Familial hemiplegic migraine (FHM) is a severe monogenic subtype of migraine with aura, characterized by the presence of hemiparesis as part of the aura phase; causative genes for FHM have been identified and functional studies have allowed to get insights into the pathophysiological mechanisms of migraine (Vecchia and Pietrobon, 2012; Pietrobon and Moskowitz, 2013; Ferrari et al., 2015; Mantegazza and Cestèle, 2018). Gain of function mutations in *CACNA1A* encoding the alpha 1a subunit of the Ca_v_2.1 (P/Q type) neuronal calcium channel are responsible for FHM-1 (Ophoff et al., 1996), loss of function mutations in *ATP1A2*, encoding the alpha 2 subunit of the Na^+^/K^+^ ATPase are responsible for FHM-2 (De Fusco et al., 2003). Moreover, the gene *SCN1A*, encoding for the voltage gated sodium channel Na_v_1.1 alpha subunit, which is the target of hundreds of epileptogenic mutations (Marini and Mantegazza, 2010; Meisler et al., 2010; Catterall, 2012; Guerrini et al., 2014), has also been identified as the target of mutations responsible for FHM-3 (Dichgans et al., 2005). Often FHM-3 patients do not show epileptic phenotypes (Mantegazza and Cestèle, 2018), and numerous studies have been undertaken to find out why a mutation in the same gene leads to different pathologies, such as epilepsies vs. FHM. We have shown that migraine mutations cause Na_v_1.1 gain of function (Cestèle et al., 2008, 2013b), a finding that has been confirmed by other studies (Fan et al., 2016). Notably, epileptogenic mutations cause the opposite effect, inducing loss of function of the channel and, because Na_v_1.1 is expressed mainly in GABAergic neurons, this leads to hypoexcitability of these neurons and reduced inhibition in neuronal networks (Yu et al., 2006; Ogiwara et al., 2007; Han et al., 2012; Hedrich et al., 2014).

Besides targeting the same gene, Na_v_1.1 epileptogenic and migraine mutations have another common point: they can cause folding/trafficking defects and, therefore, reduce the level of expression of the protein at the plasma membrane (Cestèle et al., 2013b; Bechi et al., 2015; Terragni et al., 2018). Thus far, only the mutation L1649Q responsible for FHM-3 has been shown to have this property and, importantly, we have demonstrated that under certain conditions the expression of this mutant can be partially rescued, leading to an overall effect that is consistent with gain of function (Cestèle et al., 2013b). Conversely, epileptogenic folding/trafficking defective mutants show loss of function also upon rescue, consistent with a different functional effect in comparison with migraine mutants (Rusconi et al., 2007, 2009; Bechi et al., 2015). We report here the functional study of the L1670W Na_v_1.1 mutation that has been recently identified in a Chinese family presenting with pure FHM without epileptic phenotypes (Zhang et al., 2017). We have identified the same mutation in a Caucasian American family presenting with pure FHM and, in the index patient, a remarkable clinical feature involving severe neurological deterioration after standard brain radiation for cancer treatment. We have studied the effects of this mutation both in a human cell line and in mouse neocortical neurons. We show that the mutation L1670W, similarly to L1649Q (Cestèle et al., 2013b), induces rescuable folding/trafficking defects and an overall gain of function when rescued in cell lines or when expressed in GABAergic cortical neurons. Therefore, our results confirm that FHM-3 mutations cause Na_v_1.1 gain of function and reveal that folding/trafficking defects leading to gain of function upon rescue can be a recurrent mechanism for Na_v_1.1 hemiplegic migraine mutations in different ethnic groups.

## Materials and Methods

### Genetic Testing

Genetic testing was performed by sequencing (Athena Diagnostics, Marlborough, MA, USA) to detect sequence variants in the *CACNA1A*, *ATP1A2* and *SCN1A* genes.

### Plasmids and Mutagenesis

We used the shorter splice variant isoform (-11aa) of the human Na_v_1.1 channel α subunit (GenBank database accession no. NM_006920.4) that could be the predominant Na_v_1.1 variant expressed in brain (Schaller et al., 1992; Lossin, 2009), and that we have used in numerous other studies (Cestèle et al., 2008, 2013a,b). We subcloned the Na_v_1.1 cDNA into the pCDM8 vector to minimize rearrangements (Mantegazza et al., 2005). The mutation L1670W was introduced with the Quick Change Lightning Kit (Stratagene). In order to render the hNa_v_1.1 resistant to the channel blocker tetrodotoxin (TTX), we replaced the phenylalanine at position 383 with a serine (F383S; Sivilotti et al., 1997; Bechi et al., 2012; Cestèle et al., 2013b).

### Cell Culture and Transfection

The cell line tsA-201 was maintained and transiently transfected with CaPO_4_ as already reported (Cestèle et al., 2008). Neocortical neurons were obtained from E17 OF1 mouse embryos (Charles River) and prepared as in Cestèle et al. (2013b). The dissociated neurons were plated into 35 mm dishes for patch-clamp recordings and maintained in culture at 37°C with 5% CO_2_ in Neurobasal A culture medium (Invitrogen) supplemented with B27+ (Invitrogen), glutamine 1 mM (Invitrogen), β-FGF 10 ng/ml (Invitrogen), penicillin G (50U/ml) and streptomycin 50 μg/ml (Sigma). Transfection of neurons was performed with Lipofectamin 2000 (Invitrogen) 4–5 days after the preparation and recorded 24–48 h after transfection. Cells were co-transfected with the cDNA of hNa_v_1.1 and a reporter vector expressing Yellow Fluorescent Protein (pEYFP-N1; Clontech) in order to identify the transfected cells for electrophysiological recordings. We selected neurons with fusiform bipolar morphology in order to record from GABAergic cells, as previously described (Scalmani et al., 2006; Cestèle et al., 2013b).

### Electrophysiological Recordings and Analysis

Sodium currents were recorded using the whole-cell configuration of the patch-clamp technique. Cells were recorded at room temperature (20–24°C) using a MultiClamp 700A amplifier and pClamp 10.2 software (Axon Instruments/Molecular Devices). Signal were filtered 10 kHz and sampled at 50 kHz. Electrode capacitance and series resistance were carefully compensated during the experiment. Pipette resistance was around 2–2.5 MΩ and maximal accepted voltage-clamp error was less than 2.5 mV. The remaining transient and leakage currents were eliminated using a P/4 substraction paradigm. Recording solutions for tsA-201 cells were (in mM): external solution 150 NaCl, 1 MgCl_2_, 1.5 CaCl_2_, KCl_2_ and 10 HEPES (pH 7.4 with NaOH); internal pipette solution 105 CsF, 35 NaCl, 10 EGTA, 10 HEPES and (pH 7.4 with CsOH). Recording solutions for neurons were (in mM): external solution 140 NaCl, 2 MgCl_2_, 2 CaCl_2_, 1 BaCl_2_, 1 CdCl_2_, 10 HEPES (pH 7.4 with NaOH) and TTX 1 μM; internal pipette solution (in mM): 130 CsF, 10 NaCl, 10 EGTA and 10 HEPES (pH 7.4 with CsOH). We started the recordings 5 min after obtaining of the whole cell configuration, in order to allow a complete dialysis of the cytoplasm. In the cells selected for the analysis the currents were stable also during long lasting recordings: ±10% max in comparison with the initial amplitude (in general a small run-up). Voltage dependence of activation was studied applying test pulses of 100-ms from −110 mV to +60 mV from a holding potential at −120 mV. Voltage dependence of inactivation was studied with a 100-ms prepulse at different potentials followed by a test pulse at −10 mV. The intersweep interval was 8 s. Conductance-voltage curves were derived from current-voltage (I–V) curves according to G = I/(V−V_r_), where I is the peak current, V is the test voltage, and V_r_ is the apparent observed reversal potential for tsA-201. The voltage dependence of activation and the voltage dependence of inactivation were fit to Boltzmann relationships in the form $y=1/\left(1+\exp\left((V_{\frac{1}{2}}-V)/k\right)\right)$, where *y* is normalized G_Na_ or I_Na_, ${\text{V}}_{\frac{1}{2}}$ is the voltage of half-maximal activation (V_a_) or inactivation (V_h_) and k is a slope factor; for the inactivation curve we included a baseline. Persistent current (I_NaP_) was quantified as the mean current between 40 ms and 50 ms after the beginning of the voltage step. Development of fast inactivation was studied using a prepulse of different duration at different voltage followed by a test pulse at 0 mV with an intersweep interval of 6 s. Recovery from fast inactivation was studied using a test pulse at 0 mV followed by a repolarization at −120 mV of different duration and a test pulse to 0 mV with an intersweep interval of 10 s. Development of slow inactivation was obtained using inactivating prepulses to 0 mV of increasing duration followed by repolarizations to −100 mV during 15-ms to allow complete recovery from fast inactivation and test pulses to 0 mV. Voltage dependence of development of slow inactivation was studied with 20-s-prepulse at different potentials followed by 15-ms repolarizations to −110 mV and test pulses to 0 mV. Recovery of slow inactivation was obtained using a 20-s-long inactivating prepulse to 0 mV followed by recovery interpulses at −90 mV of increasing duration and test pulses to 0 mV. Voltage dependence of recovery from slow inactivation was studied applying a 20-s-long inactivating prepulse to 0 mV followed by a 20-s recovery interpulse at different potentials and a test pulse to 0 mV. The intersweep interval for studying slow inactivation properties was 70 s. Use dependence was evaluated with trains of 200 depolarizing steps 2 ms long to 0 mV from a holding potential of −70 mV at 10 Hz, 50 Hz and 100 Hz (leak was subtracted off-line with a P/3 paradigm). Action potential clamp recordings were performed using as voltage stimulus a GABAergic neuronal discharge recorded from a fast spiking basket cell injecting a 1 s-long depolarizing current step as in Hedrich et al. (2014); the instantaneous firing frequency of the discharge was between 106 Hz and 82 Hz. Recordings were not corrected for junction potentials.

### Statistical Tests

Datasets were tested for normal distribution and the following tests were used, as indicated in the text. For normally distributed datasets, we used the unpaired Student’s *t*-test with Welch correction. For non-normally distributed datasets, we used the Mann-Whitney rank-sum test when we compared two unpaired datasets or, when we compared more than two unpaired datasets, the Kruskall-Wallis H test with Mann-Whitney post tests and Bonferroni correction for multiple comparisons. We considered the differences as statistically significant when *p* < 0.05, *p* values are provided only for significant differences. Significance is indicated in the figures as: *p < 0.05, **p < 0.01, ***p < 0.001, ****p < 0.0001. If not differently indicated, data are shown as means ± SEM, “*n*” indicates the number of cells.

## Results

### Family

**Figure 1:**
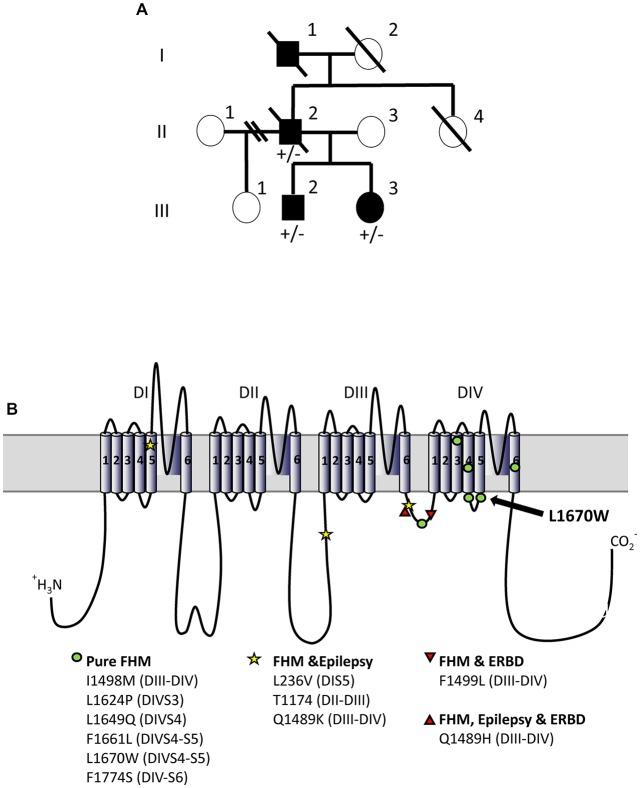
L1670W familial hemiplegic migraine (FHM-3) family. **(A)** Family pedigree. The proband is patient II-2. No genetic test was performed on patient I-1. Squares are representative of men and circles of women. Plain symbols represent affected individuals and empty symbols unaffected individuals. **(B)** Schematic representation of Na_V_1.1 alpha subunit and localization of FHM-3 mutations identified thus far; the mutation L1670W that we have studied here is highlighted with an arrow. Voltage-gated sodium channels are formed by four main domains (DI-IV), each one formed by six transmembrane segments (S1–6) connected by intracellular and extracellular loops; within each domain, S1–4 form the voltage sensing module (in which the positively charged S4 is the voltage sensor) and S5–6 form the pore module, with the intracellular loop between S4 an S5 that is the linker between the two modules. The intracellular loop between DIII-IV is the inactivation gate, which blocks the pore in the fast-inactivated states of the channels acting as a hinged lid. Slow inactivation depends on rearrangements of the pore domain. DIV is important for the coupling between activation and inactivation. Thus, most of the FHM mutations are in regions that are important for inactivation.

The index patient (II-2, Figure 1) is from a Caucasian American family and reported a history of hemiplegic migraines beginning in his teens. He had 1–2 severe attacks per year as a young man. He would typically have decreased vision on one side followed by numbness beginning in a foot that would expand to that entire side of his body. A pounding headache with photo- and phonophobia would then occur. There would be weakness and numbness on that half of the body lasting for hours, and rarely lasting as long as a day. Attacks were infrequent, often separated by many months or a year. In between attacks he had no neurological or cognitive deficits and was able to function well in his career and home life. At age 60 he was started on valproic acid and the migraine attacks and hemiplegic events essentially stopped. At age 69 he was diagnosed with limited stage small cell lung cancer and treated with CALBG-30610, cisplatin, VP-16, and standard prophylactic brain radiation. Fourteen months after cancer diagnosis he had frequent falls, worsening of memory and decreased spontaneous speech. By 16 months after diagnosis, he required a wheelchair for any mobility, had bladder incontinence, and would passively watch television all day but be unable to relate the events he watched. He continued to neurologically decline until his death 18 months after cancer diagnosis.

The deceased father (patient I-1) had a long-lasting history of hemiplegic migraine, well known to the family. Other neurological issues or cognitive deficits were not reported. He died at age 53 of “jaw cancer”.

Patient III-2 is the 29-year-old son of the index patient. He had a single febrile seizure at age 1 and was diagnosed with Attention Deficit Hyperactivity Disorder (ADHD) during childhood. He developed hemiplegic migraines at age 12. Since then events have been unpredictable, sometimes occurring several times per week and sometimes not for a year. The typical event begins with blurred vision on one side, usually the left, for 20–30 min. As the vision clears, numbness starts in the foot and spreads to that half of the body over about 30 min. Once the spread of numbness and weakness is complete he will have crushing head pain with photo- and phonophobia. The pain may last up to 4 h. He rarely has nausea or vomiting with the headaches. The affected half of the body is numb and weak for 1 h to a couple days, with his longest event persisting 1 week. During the prolonged attack he was clumsy on the left side and could not type on a computer. He shows incoordination with the affected limbs during the attacks. Between events he has no neurological symptoms. Neurological examination was normal, including mental status, cranial nerve function, strength, sensation, balance, reflexes, gait and coordination.

Patient III-3 is a 26-year-old woman, daughter of the index patient. Her medical history is notable for pseudotumor cerebri diagnosed at age 19. Pseudotumor was treated with acetazolamide for a year, and with weight loss, then resolved and remains in remission. She developed headaches at 17 years of age and has had 10 total events since then. A few headaches started with blurred vision on one side, but most had no visual aura. Headaches usually start with numbness or tingling of the foot and progress to involve the entire half of the body over 5–30 min. That half of the body feels weak as well as numb. Head pain begins during or after the spread of the numbness and weakness. Headaches are described as crushing or pounding and she seeks a quiet, dark place when they occur. Motor and sensory symptoms recover over hours to days. There is no numbness or weakness between attacks. Her neurological examination was normal, including mental status, cranial nerve function, strength, sensation, balance, reflexes, gait and coordination. MRI of the brain without contrast and MRA of the head, performed at age 21, were normal.

II-1 and III-1 were not reported to have migraine or hemiplegic attacks and were not available for genetic testing.

### Genetic Testing

Patient II-2 had sequencing of *ATP1A2* that was normal, but sequencing of *CACNA1A* could not be completed due to inadequate sample. *CACNA1A* was sequenced for patients III-2 and III-3 and showed no abnormalities. All three patients carried the heterozygous missense mutation L1670W in *SCN1A* (c.5009T>G/p.Leu1670Trp: leucine 1670 to tryptophan). This mutation has been already described in a Chinese FHM family, but functional studies have not been performed (Zhang et al., 2017). In both families, the mutation causes a pure form of familial hemiplegic migraine as it is not associated with epilepsy or Elicited Repetitive Daily Blindness (ERDB), which are co-morbidities reported in FHM patients. L1670W is localized in the linker between the voltage sensing module and the pore module of domain 4: the intracellular loop connecting S4 and S5 transmembrane segments (Figure 1B).

### The Mutation L1670W Reduces the Plasma Membrane Targeting of hNa_v_1.1, Which Is Rescued by Incubation at Lower Temperature

**Figure 2:**
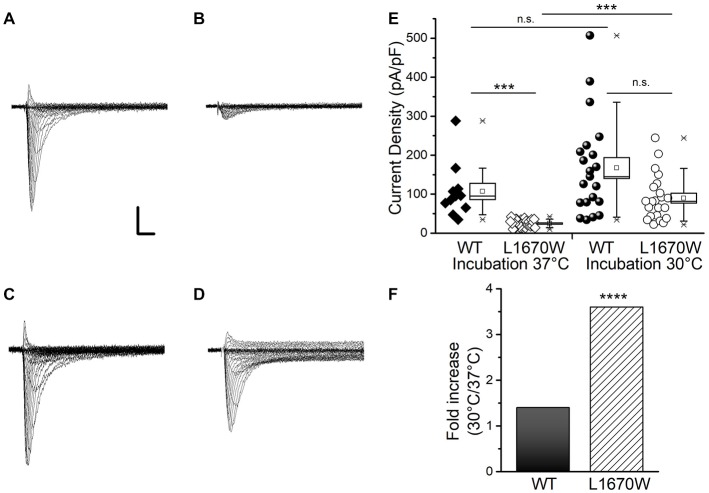
hNa_v_1.1-L1670W is a rescuable folding/trafficking defective mutant. Representative families of whole-cell sodium current traces recorded applying depolarizing steps from −70 to +60 mV in 5 mV increments (from a holding potential of −120 mV) in tsA-cells transfected with hNa_v_1.1-WT and incubated at 37°C **(A)** transfected with hNa_v_1.1-L1670W and incubated at 37°C **(B)** transfected with hNa_v_1.1-WT and incubated at 30°C **(C)** transfected with hNa_v_1.1-L1670W and incubated at 30°C **(D)**; Scale bars: 1 nA, 1 ms. **(E)** Data and box chart (the bar represents the median, the square the mean, the box the SEM and the whiskers the 10–90% range) of the mean maximum current density of hNa_v_1.1-WT incubated at 37°C (106.9 ± 21.0 pA/pF, *n* = 11), hNa_v_1.1-L1670W incubated at 37°C (24.7 ± 1.7 pA/pF, *n* = 29), hNa_v_1.1-WT incubated at 30°C (167 ± 27 pA/pF, *n* = 21), and hNa_v_1.1-L1670W incubated at 30°C (89.7 ± 12.5 pA/pF, *n* = 22); statistical comparison: hNa_v_1.1-WT-37°C vs. hNa_v_1.1-L1670W-37°C *p* = 1.2 * 10^−5^, hNa_v_1.1-WT-37°C vs. hNa_v_1.1-WT-30°C n.s., hNa_v_1.1- L1670W-37°C vs. hNa_v_1.1-L1670W-30°C *p* = 8 * 10^−7^, hNa_v_1.1-WT-30°C vs. hNa_v_1.1-L1670W-30°C n.s. (Kruskall-Wallis test followed by Mann-Whitney test with Bonferroni correction for four comparisons). ns, non significant; *p < 0.05, **p < 0.01, ***p < 0.001, ****p < 0.0001.**(F)** Fold increase in current density with incubation at 30°C.

To disclose the functional effect of the mutation hNa_v_1.1-L1670W, we transiently transfected human tsA-201 cells with WT- or L1670W-hNa_v_1.1 channels. The cells were incubated at 37°C for 24 h before to record sodium currents with the whole-cell configuration of the patch-clamp technique. Representative current traces are shown in Figures 2A,B. We observed that, in these conditions, cells expressing L1670W channels show a dramatic reduction of current amplitude in comparison with those expressing WT channels. The quantification of the current density showed a 5-fold reduction for L1670W (Figure 2E), which indicates a nearly complete loss of function of the mutant channels. This could be caused by defects of conduction or by trafficking/folding defects that reduce the targeting of the mutant channels to the plasma membrane. In order to get insights into the mechanism underlying the loss of function of hNa_v_1.1-L1670W, we incubated the transfected cells at 30°C before the recordings, a condition that is known to rescue numerous folding/trafficking defective mutant proteins, including Na_V_1.1 mutants (Bernier et al., 2004; Terragni et al., 2018). Representative current traces upon incubation at 30°C (Figure 2C for hNa_v_1.1-WT, Figure 2D for L1670W) show rescue with a substantial increase of L1670W current. Comparisons of the current densities confirmed that incubation at 30°C induced a 3.7-fold increase for L1670W, whereas the current density of WT channels was not significantly modified (Figures 2E,F). This data indicated that the incubation of the cells at 30°C can rescue L1670W mutant channels and lead to a more efficient targeting of the mutant protein at the plasma membrane, supporting the hypothesis that L1670W induces folding/trafficking-defects. Interestingly, although with incubation at 30°C there was a trend toward a reduction of L1670W current density in comparison with hNa_v_1.1-WT, the difference was not statistically significant (Figure 2E), consistent with a nearly complete rescue of L1670W in this condition.

### Effects of L1670W on hNa_V_1.1 Gating Properties in tsA-201 Cells

**Figure 3:**
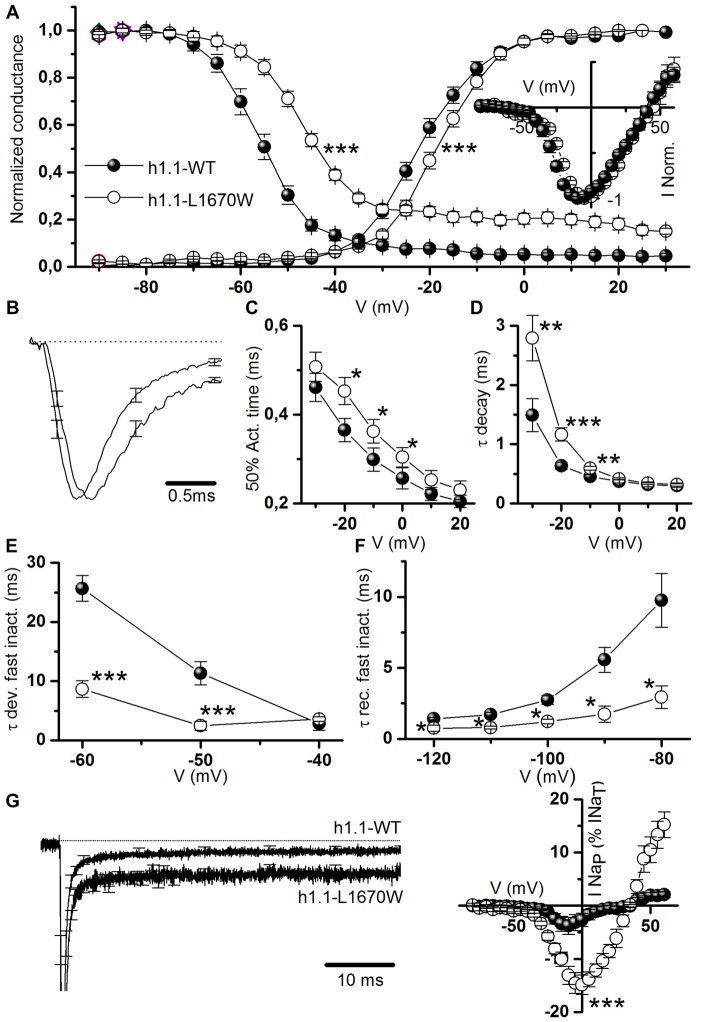
Functional effects of hNa_v_1.1-L1670W on fast gating properties upon rescue in tsA-201 cells. **(A)** Mean voltage dependence of activation and fast inactivation, lines are mean Boltzmann fits; mean parameters: voltage of half activation V_a_ and slope K_a_ for hNa_v_1.1-WT (*n* = 18), V_a_ = −21.6 ± 1.2 mV, K_a_ = 6.6 ± 0.4, hNa_v_1.1-L1670W (*n* = 22), V_a_ = −16.8 ± 1.0 mV (*p* = 0.004), K_a_ = 6.1 ± 0.5 mV; voltage of half inactivation (V_h_), slope (K_h_) and baseline for hNa_v_1.1-WT (*n* = 12), V_h_ = −55.6 ± 0.3 mV, K_h_ = 4.9 ± 0.3 mV, baseline = 0.04 ± 0.01, L1670W (*n* = 16), V_h_ = −47.3 ± 0.9 mV (*p* = 5 * 10^−5^), K_h_ = 6.1 ± 0.5 mV, baseline = 0.19 ± 0.05 (*p* = 2 * 10^−5^); Welch *t*-test. Inset: normalized current-voltage (I-V) plot of the peak transient current. **(B)** Comparison of mean normalized current traces elicited with a command step to −10 mV from a holding potential of −120 mV; the dotted line is the 0. *n* = 13 for WT and L1679W. **(C)** Time (ms) of half-activation of the current at the indicated potentials: −30 mV WT 0.46 ± 0.03, L1670W 0.51 ± 0.03; −20 mV WT 0.36 ± 0.03, L1670W 0.45 ± 0.03 (*p* = 0.02); −10 mV WT 0.29 ± 0.02, L1670W 0.36 ± 0.03 (*p* = 0.04); 0 mV WT 0.25 ± 0.02, L1670W 0.30 ± 0.02 (*p* = 0.05); 10 mV WT 0.22 ± 0.01 L1670W 0.25 ± 0.02; 20 mV WT 0.20 ± 0.01 L1670W 0.23 ± 0.02; Welch *t-test*, *n* = 13 for all groups. **(D)** Voltage dependence of the time constant (τ in ms) of the current decay (single exponential fits at the indicated potentials): −30 mV WT 1.5 ± 0.3, L1670W 2.8 ± 0.4 (*p* = 0.02); −20 mV WT 0.63 ± 0.06, L1670W 1.2 ± 0.1 (*p* = 5 * 10^−4^); −10 mV WT 0.45 ± 0.04, L1670W 0.59 ± 0.03 (*p* = 0.008); 0 mV WT 0.37 ± 0.03, L1670W 0.41 ± 0.02; 10 mV WT 0.31 ± 0.03 L1670W 0.34 ± 0.02; 20 mV WT 0.30 ± 0.03 L1670W 0.32 ± 0.02; Welch *t*-test, *n* = 13 for all groups. **(E)** τ (ms) of the development of fast inactivation at the indicated potentials: −60 mV, τ_DEV-WT_ = 25.6 ± 2.2 (*n* = 12), τ_DEV-L1670W_ = 8.6 ± 1.4 (*n* = 4; *p* = 0.009); −50 mV, τ_DEV-WT_ = 11.3 ± 2.0 (*n* = 10), τ_DEV-L1670W_ = 2.4 ± 0.8 (*n* = 7; *p* = 0.002); −40 mV, τ_DEV-WT_ = 2.8 ± 1.1 (*n* = 6), τ_DEV-L1670W_ = 3.6 ± 0.4 (*n* = 7); Mann-Whitney test. **(F)** τ (ms) of the recovery from fast inactivation at the indicated potentials: −120 mV, τ_REC-WT_ = 1.4 ± 0.2 (*n* = 7), τ_REC-L1670W_ = 0.8 ± 0.2 ms (*n* = 4); −110 mV, τ_REC-WT_ = 1.7 ± 0.2 ms (*n* = 7), τ_REC-L1670W_ = 0.79 ± 0.09ms (*n* = 3; *p* = 0.02); −100 mV, τ_REC-WT_ = 2.7 ± 0.3 ms (*n* = 8), τ_REC-L1670W_ = 1.2 ± 0.2 ms (*n* = 4; *p* = 0.014); −90 mV, τ_REC-WT_ = 5.6 ± 0.9 ms (*n* = 7), τ_REC-L1670W_ = 1.7 ± 0.6 ms (*n* = 5; *p* = 0.02); −80 mV, τ_REC-WT_ = 9.8 ± 1.9 ms (*n* = 7), τ_REC-L1670W_ = 2.9 ± 0.8 ms (*n* = 6; *p* = 0.02); Mann-Whitney test. **(G)** Left panel, comparison of the same mean normalized current traces displayed in **(B)**, but with a longer time scale in order to compare I_NaP_; right panel, mean current-voltage plots for I_NaP_ measured after 5 min from the establishment of the whole-cell configuration and expressed as percentage of the transient current (maximal I_NaP_: 4.0 ± 1.5% *n* = 21 WT, 14.9 ± 2.1% *n* = 22 L1670W; *p* = 0.002 Mann-Whitney test). Data are shown as mean ± SEM. *p < 0.05, **p < 0.01, ***p < 0.001, ****p < 0.0001.

To analyze the gating properties of WT and L1670W sodium channels, currents were recorded with the whole-cell configuration of the patch-clamp technique in tsA-201 cells previously incubated at 30°C for 24 h after transfection, to induce rescue of L1670W channels. As shown in Figure 3A, the analysis of the conductance-voltage plot highlights a significant positive shift in the voltage-dependence of activation (4.8 mV on average). Consistently, the plot of the normalized current-voltage relationships (Figure 3A, inset) shows that the maximum current was at −15 mV for the WT and at −10 mV for L1670W. In the other hand, the inactivation curve was also shifted to positive potentials (8.4 mV on average, Figure 3A) and the inactivation was not complete, as the baseline was increased 4.75-fold with L1670W, which is consistent with an increase of the persistent current (I_NaP_; see below). Notably, the effect observed on the voltage-dependence of fast inactivation is consistent with a clear gain of function induced by the mutation, whereas the positive shift of the activation curve is consistent with a loss of function.

The comparison of the kinetics of onset and of decay of the current elicited with a depolarizing step to −10 mV (Figure 3B) shows that both current onset and decay are slower for L1670W. However, quantifications over a range of potentials, displayed in Figures 3C,D, show that there is no difference between WT and L1670W channels at depolarized potentials. This is consistent with an effect due to the positive shift of the voltage dependence of activation, without a direct modification of the kinetics of onset or decay (inactivation from the open state).

The analyses of the development of fast inactivation and of the recovery from fast inactivation over a range of potentials are displayed in Figures 3E,F, respectively. Our data indicates that the L1670W mutant shows a faster development of fast inactivation from the closed states (Figure 3E) and a faster recovery from fast inactivation (Figure 3F). The effect on the development is consistent with a loss of function, whereas the effect on the recovery is consistent with a gain of function.

Mean whole-cell current traces with a longer time scale are displayed in Figure 3G and clearly show the increase of I_NaP_ induced by the L1670W mutation. Comparison over a range of potentials of the mean I_NaP_ recorded 5 min after the establishment of the whole-cell configuration (quantified as the average current between 45 ms and 55 ms after the beginning of the test pulse and expressed as percentage of maximum transient sodium current) showed that L1670W induced a 3.7-fold increase of I_NaP_ compared to the WT.

All together our data indicates that the mutation L1670W modifies several properties of the fast inactivation of Na_v_1.1 channels, inducing a positive shift of the voltage dependence of inactivation (gain of function), an acceleration of the kinetics of the development from the closed states (loss of function) and of the recovery (gain of function), as well as an increase of I_NaP_ (gain of function). This is consistent with a destabilization of the fast-inactivated state.

**Figure 4:**
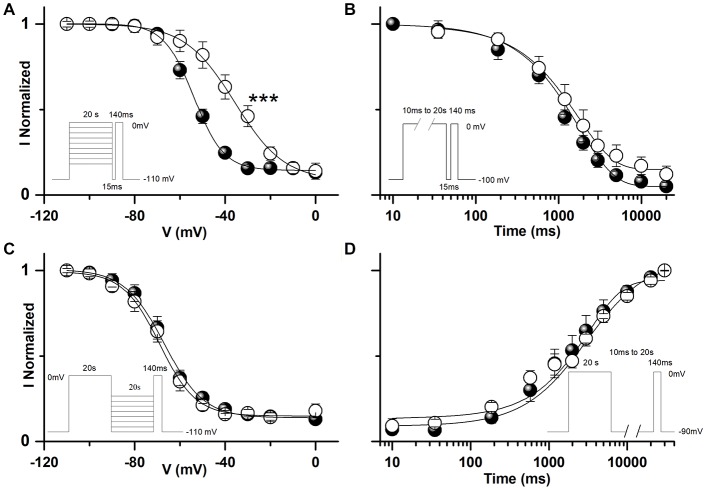
Functional effects of hNa_v_1.1-L1670W on slow inactivation upon rescue in tsA-201 cells. **(A)** Voltage dependence of development of slow inactivation; the lines are mean Boltzmann fits: mean parameters, hNa_v_1.1-WT, V_h_ = −53.5 ± 2.0 mV, K_h_ = 7.6 ± 0.9 mV, baseline 0.14 ± 0.03 (*n* = 11); hNa_v_1.1-L1670W, V_h_ = −34.8 ± 2.7 mV (*p* = 2 * 10^−4^), K_h_ = 10.0 ± 1.4, baseline 0.13 ± 0.03 (*n* = 6). ***p < 0.001. **(B)** Mean kinetics of development of slow inactivation at 0 mV for the indicated durations, the lines are mean fits of single exponentials: mean parameters, τ_DEV-WT_ = 1310 ± 184 ms, baseline 0.08 ± 0.02 (*n* = 8), τ_DEV-L1670W_ = 2070 ± 283 ms, baseline 0.15 ± 0.04 (*n* = 6). **(C)** Voltage dependence of recovery from slow inactivation; the lines are mean Boltzmann fits: mean parameters, WT, V_h_ = −66.9 ± 2.4 mV, K_h_ = 7.4 ± 1.2 mV, baseline 0.15 ± 0.03 (*n* = 8); L1670W, V_h_ = −68.3 ± 2.9 mV, K_h_ = 7.5 ± 0.9 baseline 0.15 ± 0.04 (*n* = 6). **(D)** Mean recovery at −90 mV from slow inactivation; the lines are mean fits of single exponentials: mean parameters, τ_DEV-WT_ = 3252 ± 892 ms (*n* = 6), τ_DEV-L1670W_ = 3449 ± 383 ms (*n* = 5). Welch *t*-test for all the comparisons. The insets show the voltage stimuli; the relative durations of the steps are not in scale (see the durations indicated). Data are shown as mean ± SEM.

Furthermore, we studied the effect of L1670W on the properties of slow inactivation. Our results indicate that the mutation induced a large positive shift (18.7 mV) in the curve of the voltage-dependence of the development of slow inactivation (Figure 4A). We found no significant modifications of the other properties of slow inactivation that we studied: kinetics of development Figure 4B), voltage-dependence of recovery (Figure 4C) and kinetics of recovery (Figure 4D). This effect is in agreement with a gain of function for L1670W.

Thus, analysis of the gating properties of the mutant L1670W channels compared to the wild-type highlighted that the mutation alters several parameters of the fast and slow inactivation properties of hNa_v_1.1 channels and induces a mix of loss (positive shift of the voltage-dependence of activation, faster development of fast inactivation from the closed states) and gain of function (positive shift of the voltage dependence of inactivation, increase of I_NaP_, faster recovery of fast inactivation, positive shift of the voltage dependence of the development of slow inactivation).

**Figure 5:**
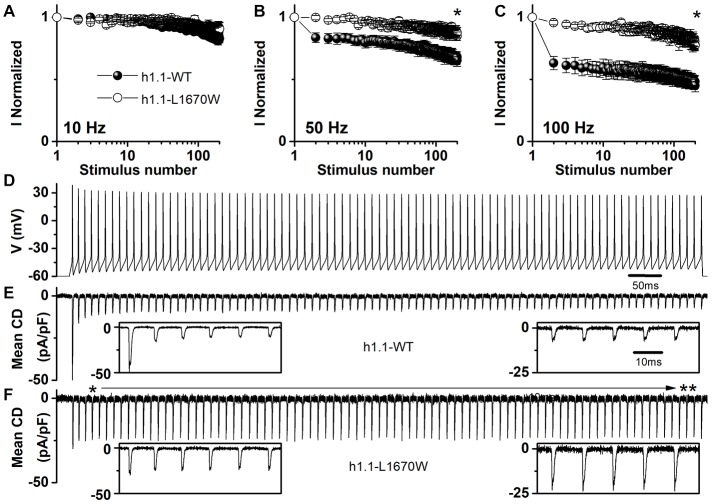
Overall effect of hNa_v_1.1-L1670W expressed in tsA-201 cells. **(A–C)** Use dependence (current normalized to the first stimulus in the train) induced by trains of 200 depolarizing steps 2 ms long to 0 mV from the holding potential of −70 mV at the frequencies of 10 Hz **(A)** 50 Hz **(B)** and 100 Hz **(C)** for hNa_v_1.1-WT (black symbols) and hNa_v_1.1-L1670W (open symbols). Comparison of the last stimulation in the train: 10 Hz WT 0.83 ± 0.06 (*n* = 5), L1670W 0.91 ± 0.05 (*n* = 5); 50 Hz WT 0.65 ± 0.05 (*n* = 5), L1670W 0.87 ± 0.04 (*n* = 5; *p* = 0.03); 100 Hz 0.45 ± 0.05 (*n* = 5), L1670W 0.77 ± 0.04 (*n* = 5; *p* = 0.02); Mann-Whitney test. *p < 0.05. **(D–F)** Action Na^+^ currents (expressed as mean current density; error bars are not shown for clarity) recorded using as voltage stimulus an action potential discharge recorded from a GABAergic fast spiking neuron in neocortical brain slices **(D)** for hNa_v_1.1-WT **(E)** *n* = 7, and hNav1.1-L1670W. **p < 0.01. **(F)** *n* = 7. Insets show the first five (left) and the last five (right) action currents; the time scale is the same for all the insets. The amplitude of the first three action currents was not different: (pA/pF) WT −49 ± 13, −16 ± 8, −14 ± 7, L1670W −30 ± 6, −27 ± 6, −26 ± 7; the first difference was observed for the 4th peak action current: WT −12 ± 4, L1670W −27 ± 6 (*p* = 0.045); comparison of the amplitude of the last five peak action currents pooled: WT 9.6 ± 1.4 (*n* = 7), L1670W 28 ± 7 (*n* = 7; *p* = 0.01); Mann-Whitney test for all the comparisons. Data are shown as mean ± SEM.

In order to disclose the effect of L1670W on use dependence, we elicited sodium currents in tsA-201 cells using trains of 2 ms-long depolarizing steps at 10 Hz, 50 Hz and 100 Hz (Figures 5A–C, respectively). Our data revealed that the mutation L1670W induces a decrease of use dependence during the train that is consistent with a gain of function as an overall effect. Furthermore, to better reproduce neuronal dynamic conditions during a discharge of action potentials, we applied as voltage command a neuronal discharge recorded in a fast-spiking GABAergic neuron (Figure 5D), in which Na_v_1.1 is particularly important, and recorded Na^+^ action currents in tsA-201 cells, quantified as current densities to normalize for the size of the cells (Figures 5E,F). Our results show a significant increase of action currents’ peak amplitude in cells transfected with the L1670W mutant compared to cells transfected with the WT. In fact, the first action current in the discharge was not significantly different, although there was a trend toward a larger peak current for the WT, but, importantly, the average of the amplitude of the last five peak action currents in the discharge was significantly larger for L1670W (2.9-fold increase, see Figure 5F).

Our results are consistent with a gain of function as overall effect of L1670W in tsA-cells, which would induce hyperexcitability of GABAergic interneurons. However, they have been obtained with incubation at 30°C, in order to rescue folding/trafficking defects of L1670W and increase its insertion into the plasma membrane. To investigate the effect of a neuronal cell background and use conditions that are more similar to pathophysiological ones, we performed experiments in transfected neocortical neurons. In fact, in our previous study of the FHM L1649Q Na_V_1.1 mutant, we observed rescue when it was expressed in neocortical GABAergic neurons without incubation at 30°C (Cestèle et al., 2013b).

### Effects of L1670W on hNa_V_1.1 in Neocortical Neurons

Primary neuronal cultures were prepared from E17 mouse embryos and neocortical neurons were transfected 4 days after the preparation with wild-type Na_V_1.1 or the L1670W mutant. In these experiments we introduced into the two clones the F383S mutation that confers resistance to the blocker TTX and does not modify functional properties, in order to block endogenous sodium currents with TTX and record selectively from the exogenous hNa_V_1.1 channels (Cestèle et al., 2013b; Bechi et al., 2015). Therefore, we performed whole-cell patch-clamp recordings 24 h–48 h after transfection from fluorescent neurons expressing the reporter YFP in the presence of 1 μM TTX.

**Figure 6:**
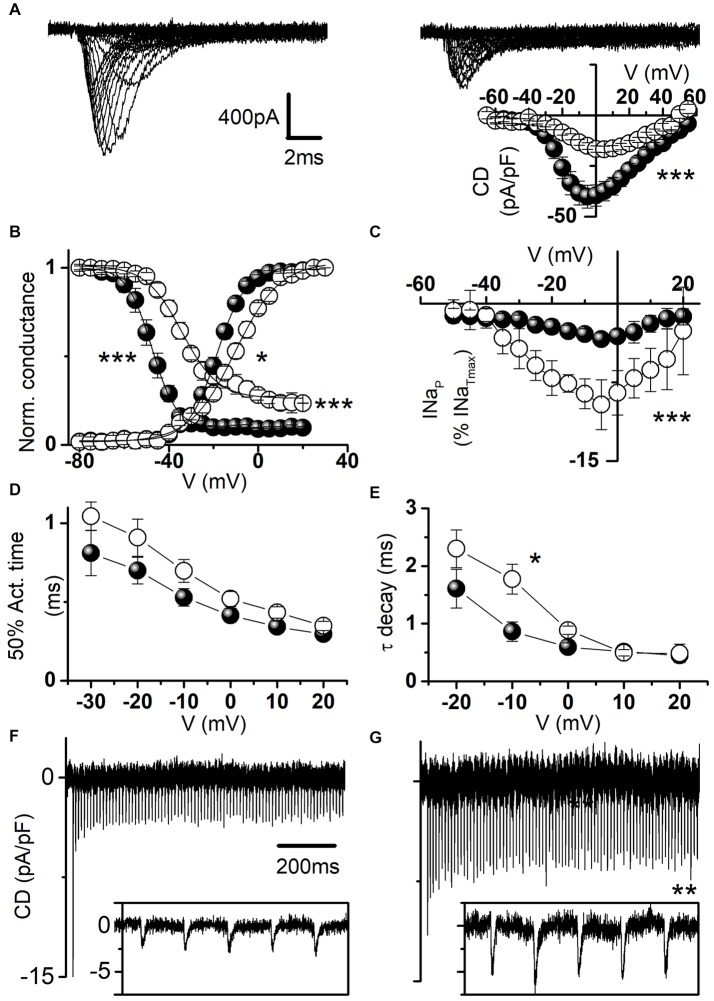
Functional effects of hNav1.1-L1670W expressed in neocortical GABAergic neurons. **(A)** Representative whole-cell sodium current traces recorded in the presence of 1 μM tetrodotoxin (TTX) from fusiform bipolar GABAergic neurons selected in primary cultures of neocortical neurons transfected with hNa_v_1.1-F383S-WT (left) or hNa_v_1.1-F383S-L1670W (right); the inset displays the mean current density-voltage plots for hNa_v_1.1-F383S-WT and hNa_v_1.1-F383S-L1670W, and the comparison of the mean maximum current density yielded: WT 41.9 ± 5.7 pA/pF *n* = 22; L1670W 19.4 ± 1.5 pA/pF *n* = 20 (*p* = 7 * 10^−4^), Mann-Whitney test. **(B)** Mean voltage dependence of activation and fast inactivation, lines are mean Boltzmann fits; mean parameters: voltage of half activation (V_a_) and slope (K_a_) for WT (*n* = 16), V_a_ = −17.5 ± 1.3 mV, K_a_ = 5.1 ± 0.3, L1670W (*n* = 15), V_a_ = −8.4 ± 1.4 mV (*p* = 10^−4^), K_a_ = 7.7 ± 0.8 mV (*p* = 0.01); voltage of half inactivation (V_h_), slope (K_h_) and baseline for WT (*n* = 15), V_h_ = −48.1 ± 1.9 mV, K_h_ = 4.3 ± 0.2 mV, baseline = 0.09 ± 0.03, L1670W (*n* = 9), V_h_ = −27.9 ± 1.5 mV (*p* = 3 * 10^−4^), K_h_ = 5.1 ± 0.7 mV, baseline = 0.26 ± 0.06 (*p* = 5*10^−4^); Welch *t*-test. **(C)** Mean current-voltage plots for I_NaP_ measured after 5 min from the establishment of the whole-cell configuration and expressed as percentage of the transient current; comparison of maximal I_NaP_: WT 5.6 ± 0.9% *n* = 21, L1670W 13.6 ± 1.9% *n* = 22, *p* = 0.005, Mann-Whitney test). **(D)** Time (ms) of half-activation of the current at the indicated potentials: −30 mV WT 0.81 ± 0.14, L1670W 1.05 ± 0.09; −20 mV WT 0.70 ± 0.08, L1670W 0.9 ± 0.1; −10 mV WT 0.53 ± 0.05, L1670W 0.69 ± 0.07; 0 mV WT 0.41 ± 0.04, L1670W 0.52 ± 0.04; 10 mV WT 0.34 ± 0.04 L1670W 0.43 ± 0.03; 20 mV WT 0.29 ± 0.03 L1670W 0.35 ± 0.03; non-significant differences, Welch *t*-test, *n* = 15 for all groups. **(E)** Voltage dependence of the time constant (τ in ms) of the current decay (single exponential fits at the indicated potentials): −20 mV WT 1.6 ± 0.3, L1670W 2.3 ± 0.3; −10 mV WT 0.86 ± 0.18, L1670W 1.77 ± 0.26 (*p* = 0.02); 0 mV WT 0.59 ± 0.07, L1670W 0.88 ± 0.07; 10 mV WT 0.51 ± 0.06 L1670W 0.49 ± 0.05; 20 mV WT 0.45 ± 0.05 L1670W 0.48 ± 0.16; Welch *t*-test, *n* = 15 for all groups. Whole-cell action sodium currents (expressed as mean current density; error bars are not shown for clarity) recorded as in Figures 5D–F using as voltage stimulus an action potential discharge recorded in a neocortical mouse brain slice from a GABAergic fast spiking neuron for WT (*n* = 11; **F**) and L1670W (*n* = 9; **G**): comparison of the amplitude of the first peak action current in the discharge, WT 19.6 ± 2.9 pA/pF; L1670W 12.7 ± 1.8 pA/pF; the first action current with amplitude statistically different was the 3rd in the discharge: WT 5.9 ± 1.5 pA/pF; L1670W 10.5 ± 1.4 pA/pF (*p* = 0.048); comparison of the average of the last five peak action currents (pooled) in the discharge, WT 5.2 ± 0.8 pA/pF; L1670W 9.0 ± 1.0% pA/pF (*p* = 0.01); Welch *t*-test for all the comparisons. Data are shown as mean ± SEM.

Figure 6A displays representative sodium current traces and shows that hNa_v_1.1-L1670W mutants are partially rescued when expressed in neocortical neurons incubated at 37°C. Thus, incubation at lower temperature is not necessary for partially rescuing L1670W in a GABAergic neuron cell background, similarly to the results that we obtained with the FHM L1649Q mutation (Cestèle et al., 2013b). However, the current density of L1670W was significantly reduced by about 50% in comparison with that recorded with WT channels (Figure 6A, inset), differently than in tsA-201 cells incubated at 30°C, in which L1670W current density showed a trend towards reduction that did not reach statistical significance (Figure 2). Thus, we next investigated whether L1670W modifies hNa_v_1.1 gating properties in neurons similarly to tsA-201 cells and whether the overall effect is gain of function also in neurons, despite the reduction in current amplitude. As shown in Figure 6B, the analysis of the voltage dependence of activation and of fast inactivation yielded results that are similar to those obtained in tsA-201 cells (Figure 3A). In fact, the conductance-voltage plot showed a significant positive shift in the voltage-dependence of activation (9 mV on average), the inactivation curve showed a larger positive shift (20 mV on average) and the inactivation was not complete (the baseline was increased 2.9-fold). Consistently and similarly to the data obtained with tsA-cells, L1670W increased I_NaP_ by 2.4-fold, as shown by the mean current-voltage plots for I_NaP_ expressed as percentage of the transient current (Figure 6C). The quantification of the current onset kinetics over a range of potentials (Figure 6D) showed a trend that was similar to the effect observed in tsA-201 cells, but it did not reach statistical significance. Similarly, the kinetics of current decay showed a trend that was similar to the effect observed in tsA-201 cells, but only the point at −10 mV was significantly different (Figure 6E). We did not study slow inactivation properties because the protocols are challenging for recordings from neurons in primary culture. These results show that the effect on gating properties is similar in GABAergic neurons and tsA-201 cells, with some effects clearly inducing a gain of function and others clearly inducing a loss of function.

We investigated the overall effect of the mutation in transfected GABAergic neurons by action potential clamp experiments. Figures 6F,G show action currents elicited expressing the WT or the L1670W mutant, respectively, and applying a fast-spiking neuron discharge as voltage command. Comparing the amplitudes of the action currents expressed as current density, we found that the first current in the discharge showed a trend towards larger amplitude for the WT, which did not reach statistical significance, whereas the average of the peak amplitude of the last five currents in the discharge showed a 1.7-fold increase for L1670W, consistent with an overall gain of function.

This data indicates that in a GABAergic neuronal cell background the conditions needed to allow the expression of the hNa_v_1.1-L1670W mutant at the plasma membrane are present. The effects observed on gating properties are in agreement with those that we obtained in tsA-201 cells and the action potential clamp experiments show that the overall effect of hNa_v_1.1-L1670W is gain of function.

## Discussion

We report the functional characterization of the L1670W hNa_v_1.1/*SCN1A* missense mutation, which we identified in a Caucasian American family presenting with FHM, and that was previously found in a Chinese family with pure FHM (Zhang et al., 2017). None of the patients of the two families reported epilepsy or other clinical issues that have been associated to Na_v_1.1 hemiplegic migraine mutations, such as cerebellar ataxia or ERDB. Thus, L1670W is a recurrent mutation identified in different ethnic groups causing pure hemiplegic migraine. Notably, our index patient had progressive cognitive impairment after receiving standard whole brain radiation for cancer treatment, at a dose that is usually well tolerated. In our knowledge, this is the first report of a *SCN1A* positive patient showing this clinical feature, Interestingly, *SCN1A* is among the genes up-regulated after low dose brain radiation (Lowe et al., 2009), but it has never been investigated whether brain radiation has higher risk for patients with FHM or other *SCN1A* mutations. The results of our study show that the mutation in some conditions causes an almost complete loss of function of Na_v_1.1 because of folding/trafficking defects. However, the mutant is partially rescued by incubation at lower temperature or by expression in neurons (the latter is a condition that more closely mimics *in vivo* pathophysiological conditions) and, upon rescue, the modifications of gating properties induced by L1670W lead to an overall gain of function, similarly to other FHM-3 mutations. Interestingly, Na_v_1.1 gain of function and radiation-induced upregulation of *SCN1A* might act together to cause brain injury upon radiation therapy. Importantly, we report a single case with this clinical feature and the effect of the radiation therapy may be just coincidental, but it will be important to shed light on this issue in future studies.

Na_v_1.1 is the target of hundreds of mutations implicated in different epilepsy phenotypes (Marini and Mantegazza, 2010; Meisler et al., 2010; Catterall, 2012; Guerrini et al., 2014). Migraine and epilepsy share some pathologic features. They are episodic disorders in which patients are affected sporadically with paroxysmal attacks. Moreover, aberrant neuronal excitability is implicated in the pathological mechanisms of both diseases. However, of the 11 Na_v_1.1 mutations linked thus far to hemiplegic migraine, nine cause hemiplegic migraine without epilepsy (Mantegazza and Cestèle, 2018), consistently with different pathological mechanisms between epileptogenic and migraine Na_v_1.1 mutations. Thus, the comparison of the functional effects of L1670W and of other Na_v_1.1 migraine mutations with those of Na_v_1.1 epileptogenic mutation can contribute to shed light on the differential pathological mechanisms of migraine and epilepsy.

Numerous functional studies and gene-targeted mouse models have shown that epileptogenic mutations cause loss of function of Na_v_1.1 (Guerrini et al., 2014), whereas we and others have shown that hemiplegic migraine Na_v_1.1 mutations cause gain of function of the channel (Cestèle et al., 2008, 2013a,b; Fan et al., 2016). We confirm here that also L1670W causes an overall gain of function. Na_v_1.1 is expressed mainly in GABAergic neurons; thus, epileptogenic mutations lead to hypoexcitability of these neurons and reduced inhibition in cortical networks, consistent with the epileptic phenotype observed in patients and gene targeted mice (Yu et al., 2006; Ogiwara et al., 2007; Han et al., 2012; Hedrich et al., 2014). The pathologic mechanism of FHM Na_v_1.1 mutations is more counterintuitive. We have shown that FHM-related Na_v_1.1 gain of function induces hyperexcitability of transfected GABAergic neurons (Cestèle et al., 2008, 2013b). This effect is confirmed for L1670W by the action potential clamp experiments that we have performed applying as voltage command a GABAergic neuronal discharge (recorded from a fast spiking neuron), which elicits larger sodium currents in both tsA-201 cells and GABAergic neurons transfected with L1670W in comparisons with Na_v_1.1-WT.

We have hypothesized that hyperexcitability of GABAergic neurons could cause extracellular K^+^ and neurotransmitter accumulation, inducing neuronal depolarization and initiation of cortical spreading depression, a wave of transient network hyperexcitability leading to a long-lasting depolarization block of neuronal firing that is a proposed pathological mechanism of migraine (Cestèle et al., 2008, 2013b; Mantegazza and Cestèle, 2018). As in other episodic disorders, migraine patients show symptoms during paroxysmal attacks. The attacks are sporadic in FHM3: in general 3/4 per year, as in our family. It is hypothesized that mutations involved in episodic disorders do not directly trigger attacks, but lower the threshold for trigger factors (Pietrobon and Moskowitz, 2013). FHM mutations could lower the threshold for cortical spreading depression triggers. It is interesting to note that, as shown by our use dependence experiments (Figures 5A–C), L1670W induces a net increase in current only when channels are activated at high frequency. This feature has been observed also for other FHM-3 mutations (Cestèle et al., 2008, 2013b; Fan et al., 2016) and is consistent with dysfunctions induced mainly in some functional states (when neuronal networks are challenged and neurons fire at high frequency).

As our present and previous studies show, one striking functional effect that epileptogenic and migraine Na_v_1.1 missense mutations can have in common is the rescuable reduction of current density, which depends on reduced channel density in the plasma membrane (Bechi et al., 2015). This effect can be caused by folding or trafficking defects (including protein instability in the plasma membrane). Although we have not investigated detailed mechanisms, the rescue induced by incubation at low temperature is shared by numerous folding defective mutants. They are recognized as incorrectly folded proteins by the quality control system of the endoplasmic reticulum and degraded, but they can be rescued by physical conditions (e.g., low temperature) or molecular interactions (Bernier et al., 2004; Terragni et al., 2018). Interestingly, the overall effect of epileptogenic hNa_v_1.1 mutations remains loss of function also upon rescue (Rusconi et al., 2007, 2009; Cestèle et al., 2013b). We have shown here that also L1670W induces rescuable folding/trafficking defects, but when rescued its overall effect becomes gain of function, similarly to the L1649Q FHM-3 mutant that we have previously studied (Cestèle et al., 2013b). Therefore, L1670W is a further FHM-3 mutant showing this feature, which may be a common mechanism for FHM-3 mutations. Interestingly, both L1649Q and L1670W showed an almost complete loss of function when expressed in tsA-201 cells, whereas expression in neurons induced a partial rescue. However, effects on gating properties were similar both in tsA-201 cells and in neurons. Overall, the differential effect upon rescue is consistent with the hypothesis that epileptogenic Na_v_1.1 mutants cause loss of function, whereas the FHM-3 ones cause gain of function.

Notably, the differential cell background between tsA-201 cells and neurons does not provide the same rescuing conditions but does not modify the effects on gating properties. This discrepancy between cell lines and neurons should be taken into account when functional studies are performed, because lack of rescue would lead to the identification of loss of function effects for mutants that instead induce gain of function. In our experiments with cell lines, we have not co-expressed interacting proteins because their effect, including that of accessory β subunits, is mutation-dependent (Cestèle et al., 2013b; Bechi et al., 2015) and it is not possible to test all the interacting proteins identified and their combinations. On the other end, neurons provide a more physiologic cell background. Although transfected cells are reduced experimental systems, they allow screens of numerous mutations and investigations of detailed functional properties that are much more difficult to perform with other systems (Mantegazza et al., 2010). The identification of the best conditions for their use (e.g., using in parallel cell lines and neurons) can allow obtain reliable results.

Our functional analysis of hNa_v_1.1-L1670W points out that the gain of function is mainly caused by the destabilization of the inactivated states, both in tsA-201 cells and in GABAergic neurons (positive shift of the voltage dependence of fast inactivation, increase of I_NaP_, faster development and faster recovery of fast inactivation and positive shift of the voltage dependence of development of slow inactivation). We observed an analogous effect for L1649Q, the other folding/trafficking defective FHM-3 Na_v_1.1 mutant that we have studied (Cestèle et al., 2013b), with similar modifications of fast inactivation and increase of I_NaP_. In particular, increase of I_NaP_is a common effect that we have observed in FHM migraine mutations (Cestèle et al., 2008, 2013a,b). Interestingly, L1670W and L1649Q show distinct effect on slow inactivation, because L1670W modifies mainly the voltage dependence of the development whereas L1649Q modifies mainly the other properties of slow inactivation, including kinetics. These differences in functional effects may be explained by the fact that they are localized in different structural region of the channel. Both L1670W and L1649Q are in the domain IV (DIV) of Na_v_1.1 (Figure 1B), in the intracellular loop between transmembrane segments S4 and S5 and in the transmembrane segment S4 (the positively charged voltage sensor), respectively. Notably, most of the Na_v_1.1 hemiplegic migraine mutations described so far in the literature are in DIV or in the intracellular loop between DIII and DIV, and all the mutations causing pure FHM are in DIV, except the mutation I1498M that is in the inactivation loop (Figure 1). These are regions directly involved in inactivation of the channel. The loop between DIII and IV is the inactivation loop, which acts as a hinged lid that blocks the pore when the channel is in fast inactivated states (Catterall, 2014). DIV is essential for activation-inactivation coupling, because DIV voltage sensor activates (S4 moves outward) after the voltage sensors of the other domains, inducing a distinct pore conformation that leads to fast inactivation (Goldschen-Ohm et al., 2013). The intracellular loop between transmembrane segments S4 and S5, in which L1670W is located, is the functional linker between the voltage sensing modules and the pore modules. The comparison of the crystal structures of channels in different states has shown that the outward movement of S4 driven by depolarization induces a movement of the S4–S5 linker, which causes a bending and twisting motion of S5 and S6, that open the pore and, in domain IV, to the establishment of a pore conformation that leads to inactivation (Catterall, 2014).

Interestingly, another FHM-3 mutation (F1661L) has been identified in DIV S4-S5 loop, but functional studies have not been performed (Weller et al., 2014). However, there are other Na_V_1.1 missense mutations in these regions: 14 mutations of DIV-S4 and 14 of DIV S4-S5 linker have been identified in epileptic patients^1^. We have performed the functional study of the S4-S5 linker M1664K mutation, which caused a complete loss of function of hNa_V_1.1, because the mutant was expressed but not targeted to the plasma membrane and the rescuing approaches that we have tested, including incubation at lower temperature, were unsuccessful (Bechi et al., 2015). Thus, rescuing properties are different also for mutants that are at very close locations in the same structural region. The functional study of another DIV S4-S5 mutation, F1661S, showed reduced current density, impaired recovery from slow inactivation and increased I_NaP_ (Rhodes et al., 2004). DIV S4 mutations that have been functionally studied are R1648H (which facilitated inactivation; Tang et al., 2009; Hedrich et al., 2014), R1648C (which induced a negative shift of the fast inactivation curve, a positive shift of the activation curve and an increase of I_NaP_; Rhodes et al., 2005), I1657M (which induced a negative shift of the fast inactivation curve and a positive shift of the activation curve; Lossin et al., 2003), and R1657C (which induced reduced current density, a positive shift of the activation curve and accelerated recovery from fast and slow inactivation; Lossin et al., 2003). Therefore, the overall effect of these epileptogenic mutations is consistent with loss of function and, in general, the final effect of a mutation does not directly depend on the structural region in which it is located.

Altogether, we have characterized the functional effect of the sixth mutant causing pure FHM-3. The loss of function caused by a major reduction of current density transformed into overall gain of function when the mutant is rescued suggests a recurrent mechanism for FHM-3 mutations. Therefore, our study provides important insights into the mechanism of FHM-3 mutations and can contribute to the understanding of differential pathophysiological mechanisms in comparison with epileptogenic mutations.

## Author Contributions

SD performed electrophysiological experiments and analysis, and participated to the writing of the manuscript. EL performed the clinical part of the study and participated in the writing of the clinical/genetic results. SM supervised the genetic analysis and participated in the writing of the genetic results. NL performed DNA sequencing. MM supervised the electrophysiological experiments and their analysis and participated in the writing of the manuscript. SC performed molecular biology experiments and preliminary electrophysiological experiments, performed data analysis and wrote the manuscript.

## Conflict of Interest Statement

The authors declare that the research was conducted in the absence of any commercial or financial relationships that could be construed as a potential conflict of interest.

## Footnotes

^1^http://www.caae.org.cn/gzneurosci/scn1adatabase/index.php
